# Association of Home Food Availability with Prediabetes and Diabetes among Adults in the United States

**DOI:** 10.3390/nu12051209

**Published:** 2020-04-25

**Authors:** Jennifer R. McAtee, Meng-Hua Tao, Christian King, Weiwen Chai

**Affiliations:** 1 Department of Nutrition and Health Sciences, University of Nebraska-Lincoln, 1700 N 35th Street, Lincoln, NE 68583, USA; jennifer.mcatee@huskers.unl.edu; 2 Department of Biostatistics and Epidemiology, University of North Texas Health Science Center, 3500 Camp Bowie Boulevard., Fort Worth, TX 76107, USA; Menghua.Tao@unthsc.edu; 3 Department of Health Management and Informatics, University of Central Florida, 500 W Livingston Street, Suite 402G, Orlando, FL 32801, USA; Christian.King@ucf.edu

**Keywords:** home food availability, diabetes, prediabetes, healthy food, unhealthy food, healthy food availability

## Abstract

This study examined associations of home food availabilities with prediabetes and diabetes among 8929 adults (20–70 years) participating in 2007–2010 National Health and Nutrition Examination Surveys. Odds ratios (ORs) and 95% confidence intervals (95% CIs) were estimated by logistic regression. Relative to non-diabetic participants (individuals without diabetes or prediabetes), prediabetes participants were associated with lower availabilities of green vegetables (OR = 0.82; 95% CI = 0.73–0.91; *p* = 0.0006) and fat-free/low-fat milk (OR = 0.80, 95% CI = 0.65–0.89; *p* = 0.001) and higher sugary drink availability (OR = 1.24, 95% CI = 1.04–1.48; *p* = 0.02), adjusting for age, sex, and ethnicity (Model 1). The associations remained significant for vegetables (*p* = 0.005) and fat-free/low-fat milk (*p* = 0.02) adjusting for additional confounders (body mass index, education, Model 2). Adjusting for dietary components did not change the above results (in model 2) significantly. Participants with high healthy food availability scores had approximately 31% reduction (*p =* 0.003) in odds of prediabetes compared to those with low scores in Model 1. No associations were detected for diabetes except for fat-free/low-fat milk availability, for which an inverse association was observed in Model 1 (OR = 0.80, 95% CI = 0.65–0.99; *p* = 0.04). The results show prediabetes participants had lower availability of healthy foods and higher availability of unhealthy foods, suggesting the need to improve healthy food availability at home for this population.

## 1. Introduction

Diabetes is the seventh leading cause of death in the United States. In 2015, approximately 30.3 million (9.4% of the population) Americans had diabetes [1]. In addition, approximately one third of the U.S. adults have prediabetes [1], which is characterized by blood glucose levels that are elevated but not high enough to be diagnosed as diabetes [1]. However, only eleven percent of these individuals reported being aware of their prediabetes status [1]. Without proper treatment and prevention efforts, a majority (~70%) of persons with prediabetes will eventually become diabetic [2].

To date, the prevention of type 2 diabetes has mainly focused on behavioral modification and weight management, as causes of the disease are attributed largely to obesity, lack of physical activity, and poor dietary patterns [3,4,5,6]. Home food availability defined as the presence or absence of healthy and unhealthy food items at home is an important avenue to investigate since it may reflect people’s food choices and their dietary intake [7,8,9]. It has been reported the availability of unhealthy foods in the home was significantly associated with energy intake in both adults and their adolescent children [7]. Among older adults (50 years old), home “obesogenic foods’” scores were predictive of intakes of nutrients such as saturated fat, sugar, and fiber [9]. In addition, previous work found that obese individuals had fewer healthy foods available in their homes than that of non-obese participants [10]. Considering the established relationship between diet, obesity, and metabolic diseases such as type 2 diabetes [3,4,5,6,11,12], it is likely that the home food environment may, in turn, contribute to the prevalence and development of prediabetes and/or type 2 diabetes. Research has shown the availability of healthy foods at home was associated with lower rates of prediabetes and diabetes among adolescents [13]. To our knowledge, no studies have examined these associations in adults using a nationally representative sample. Further, the home food environment and dietary habits could differ between persons with type 2 diabetes and those with prediabetes given the consideration that many prediabetes individuals are not aware of their prediabetes status [1]. Thus, when assessing the relationship between home food availability and metabolic disease conditions, it is important to distinguish individuals with prediabetes from those with diabetes. Therefore, the primary objective of this study was to examine associations of the availabilities of individual healthy (fruits, dark green vegetables, and fat-free/low-fat milk) and unhealthy foods (salty snacks and sugary drinks) at home as well as overall home healthy food availability with the presence of prediabetes or diabetes among adults aged 20–70 years using a nationally representative sample from the 2007–2010 National Health and Nutrition Examination Surveys (NHANES). In addition, we examined whether the above associations, if present were independent of individuals’ dietary intake.

## 2. Materials and Methods

### 2.1. Study Population

NHANES is an ongoing program of studies intended to assess the health and nutritional status of approximately 5000 adults and children in the United States each year. NHANES uses a complex, multistage, probability sampling design to select participants who are geographically dispersed and representative of the civilian, noninstitutionalized US population [14].

The 2007–2010 data were used since questions regarding home food availability were only assessed in 2007–2010 NHANES surveys. There were 10,044 age-eligible respondents (20–70 years). Sequential exclusions included missing data for food availability, hemoglobin A1C (HbA1C) values, and response to diabetes diagnosis questions, with overlap from participants who were missing data in more than one category (N = 919). Additionally, individuals who were categorized as non-diabetic (participants without the presence of diabetes or prediabetes) but reported taking insulin or diabetes medications (N = 34), as well as participants who reported being pregnant (N = 114), were excluded from analysis. Furthermore, we also excluded participants who had missing data on key covariates (age, sex, race/ethnicity, body mass index [BMI], education) (N = 48). There were no statistically significant differences in sociodemographic characteristics between participants with and without these missing values. The final analytic sample included 8929 adults including 4448 men and 4481 women.

### 2.2. Relevant Measures and Independent/Dependent Variables

#### 2.2.1. Home Food Availability of Individual Food Items

The home food availability questions from NHANES Consumer Behavior questionnaire measured the frequency of availability of certain healthy and unhealthy food and beverage items in the home [14]. Although the questions did not provide a specific reference evaluation period, it can be assumed that participants were asked “in general” or on a “typical day/week/month”. Items considered “healthy” were fruits (fresh, dried, canned, and frozen fruits), dark green vegetables (fresh, dried, canned, and frozen vegetables), and fat-free/low-fat milk (1%, skim or fat-free; excluding 2%). Items considered “unhealthy” were salty snacks (such as chips and crackers; excluding nuts) and sugary drinks (soft drinks, fruit-flavored drinks, or fruit punch; excluding diet drinks, 100% juice, or sport drinks). The classification of “healthy” and “unhealthy” foods and beverages were derived from previous home food inventory tools and literature [7,15].

A five-point scale describing how often the food items were available in the home (always, most of the time, sometimes, rarely or never available) was used for survey responses and was coded on a scale of 1–5 with “1” referring “always” and “5” referring “never available” [14]. We further classified the above scale into two categories stressing the importance of contrasting the two values reflecting the frequency of home food availabilities: (1) “high availability” group (recoded as “1”) for participants who responded with “always” or “most of the time available”; and (2) “low availability” group (recoded as “2”) for participants who responded with “sometimes”, “rarely”, or “never available”.

#### 2.2.2. Overall Healthy Food Availability at Home

Using the responses to the questions of availabilities of the aforementioned five food items from NHANES Consumer Behavior questionnaire [14], a scoring system for the overall home healthy food availability [HFA] was created by summing the number of positive responses from 0 to 5. A participant scored 5 (maximum points) if he/she had all the positive responses (i.e., high availability for fruits, vegetables, and fat-free/low-fat milk; low availability for salty snacks and sugary drinks). Scores 0–4 were given corresponding to 0–4 (out of 5) positive responses. We further combined the scores into high, medium, and low categories. A high score (overall healthy) was defined as having an HFA score of 5 or 4; a medium score (borderline) was defined as having an HFA score of 3 or 2; and a low score (overall unhealthy) was defined as having an HFA score of 1 or 0. Unhealthy items (salty snacks, sugary drink) were reversely scored. High, medium, and low categories were recoded as “3”, “2” and “1”, respectively.

#### 2.2.3. Prediabetes and Diabetes Status

The presence of diabetes was defined as having any of the following: (1) Hemoglobin A1c (HbA1c) concentration ≥6.5% [16]; or (2) self-report of diabetes diagnosis (yes on the question “did your doctor tell you that you have diabetes?”). Prediabetes was defined as: (1) HbA1c between 5.7% to 6.4% [16]; and (2) participants reporting “no” on the diabetes diagnosis question. Participants were categorized as non-diabetic (without the presence of diabetes or prediabetes) if they reported “no” on their diabetes status, were not taking any diabetes medications, and had a HbA1c laboratory measure lower than 5.7%.

#### 2.2.4. Nutrient Intake Assessments

The NHANES dietary interview component gathers detailed dietary intake from participants. On two separate occasions, participants reported their food and beverage intake over the past 24 h using the USDA’s Automated Multiple-Pass Method [17,18,19]. The two 24-h recalls were conducted in NHANES 2007 to 2010. The first dietary recall was collected in person by trained interviewers in NHANES Mobile Exam Center (MEC) and the second dietary recall was completed by trained interviewers via telephone 3–10 days after the MEC interview [18]. The data collected from each participant’s two 24-h recall interviews were coded and linked to a database of foods and beverages and their nutrient compositions. The database was used to estimate the types and amounts of food and beverages (including water) consumed, as well as to estimate energy, nutrients, and other components from those food and beverage items. Dietary recall data were collected on both weekdays and weekend days. Dietary intakes can vary by day of the week. Thus, special dietary weights were computed for each survey cycle that adjust for the differences in the proportion of recalls on weekdays compared with weekend days [17,18]. The current study used both dietary recall interviews to establish a mean estimate of daily dietary intake for total energy, carbohydrate, protein, total fat, saturated fat, dietary fiber, and total sugar.

### 2.3. Statistical Analysis

The “Survey” procedure in SAS 9.4 software (SAS Institute, Cary, NC, USA) was used to estimate variance after incorporating the weights for the sample population in NHANES [20]. Participants’ characteristics and dietary intake of total energy and major nutrients (carbohydrate, protein, total fat, saturated fat, dietary fiber, and sugar) were compared between diabetes, prediabetes, and non-diabetic participants using *t* test for continuous variables and chi-square test for categorical variables. Odds ratios (ORs), 95% confidence intervals (95% CIs) and *p _trend_* for associations of home food availabilities with the presence of diabetes or prediabetes were estimated using logistic regression accounting for survey design and weights. The models were adjusted for age (continuous), sex, and race/ethnicity (whites, blacks, Hispanics, or other race/ethnicity) (Model 1), and additionally adjusted for BMI (continuous) and education (non-college graduates or college graduates) (Model 2). We also considered smoking status and physical activities as covariates but did not include it in the final models because it did not alter the estimates substantially. Previous research found indicators of socioeconomic status including both monetary factors such as income/poverty [21,22] and food security [23] and non-monetary factors such as education [22] can be important predictors of home food availability. However, comparing to economic resources such as income to poverty ratio, an individual’s education background appeared to be a more significant predictor for the availability of both healthy and unhealthy foods at home from NHANES [22]. Thus, to avoid over-adjustment in this cross-sectional analysis, we only included the most relevant socioeconomic indicator (college education) as a covariate in the full models (Model 2).

To test whether associations of home food availabilities with diabetes or prediabetes were independent of individuals’ dietary intake, our models were further included dietary variables as covariates (Model 3). Thus, Model 3 was adjusted for all the covariates in Model 2 as well as dietary variables including total energy, carbohydrate, sugar, fat (total) and saturated fat. Dietary two-day sample weights were applied in the analyses. All of the reported *p*-values were two-tailed, and statistical significance was set at 0.05. Since the NHANES database is publicly available, no Institutional Review Board approval was required.

## 3. Results

The current study included 8929 participants (4448 men and 4481 women) aged 20–70 years, of which 1197 participants (13.4%) had diabetes and 2075 (23.2%) had prediabetes. Study participants with prediabetes or diabetes were more likely to be older and blacks, have higher BMI and lower education achievement compared to participants without prediabetes or diabetes (non-diabetic participants). Moreover, participants with diabetes were less likely to be current smokers than those with prediabetes or non-diabetic individuals (Table 1). With respect to dietary intake, diabetes group consumed less total energy, carbohydrate, and sugar compared to non-diabetic or prediabetes group, while no significant differences in dietary intakes of major nutrients and total energy were observed between non-diabetic and prediabetes groups (Table 1).

Results of the availabilities of individual food items at home with the presence of prediabetes or diabetes were shown in Table 2. Compared to non-diabetic individuals, prediabetes participants were more likely to have low availabilities of dark green vegetables (OR = 0.82; 95% CI =0.73–0.91; *p* = 0.0006) and fat-free/low-fat milk (OR = 0.80; 95% CI = 0.65–0.89; *p* = 0.001), and high availability of sugary drinks (OR = 1.24; 95% CI = 1.04–1.48; *p* = 0.02) in model 1 adjusting for age, sex, and ethnicity. The associations remained statistically significant for green vegetables (OR = 0.86; 95% CI = 0.78–0.95; *p* = 0.005) and fat-free/low-fat milk availabilities (OR = 0.82; 95% CI = 0.69–0.97, *p* = 0.02) in Model 2 adjusting for additional confounders such as BMI and education. Home food availability was not associated with the presence of diabetes with the exception of fat-free/low-fat milk as an inverse association was observed for the availability of this food item in Model 1 (OR = 0.80, 95% CI = 0.65–0.99; *p* = 0.04).

We also assessed whether the associations of home food availabilities with prediabetes or diabetes were influenced by an individual’s dietary intake with further adjustment for dietary variables (Model 3) in addition to the covariates included in Model 2. The associations with prediabetes remained significant for dark green vegetables (OR = 0.87; 95% CI = 0.77–0.99; *p* = 0.03) and fat-free/low-fat milk (OR = 0.79; 95% CI = 0.67–0.93; *p* = 0.007) availabilities after adjusting for dietary intakes of total energy, carbohydrate, sugar, fat (total) and saturated fat. There was a borderline association between sugary drink availability and prediabetes in Model 2 (OR = 1.17; 95% CI = 0.99–1.37; *p* = 0.06) and this borderline association appeared to be attenuated after accounting for above dietary factors (*p* = 0.26) (Table 2).

Likewise, overall home healthy food availability (HFA) scores were inversely associated with the presence of prediabetes in Model 1 adjusting for age, sex, and ethnicity (*P_trend_* = 0.004). Participants with high HFA scores (overall healthy) had approximately 31% reduction in odds of prediabetes compared to those with low scores (overall unhealthy) (OR = 0.69; 95% CI = 0.55–0.87; *p* = 0.003). The association was attenuated in Model 2 adjusting for additional confounders including BMI and education (*P_trend_* = 0.07) as well as in Model 3 (*P_trend_* = 0.08) adjusting for confounders in Model 2 plus dietary components (total energy, carbohydrate, sugar, fat [total], saturated fat). However, there was still a significant reduction (22%) in odds of prediabetes for those with high HFA scores versus those with low HFA scores (OR = 0.78; 95% CI = 0.61–0.99; *p* = 0.04) when further adjusting for dietary components in Model 3 (Table 3). In addition, among participants with diabetes (N = 1197), 27.2% were characterized as having high HFA scores (overall healthy), 63.0% were characterized as having medium HFA scores (borderline) and 9.8% were characterized as having low HFA scores (overall unhealthy). The corresponding distributions were 28.3%, 60.5% and 11.2% for participants with prediabetes (N = 2075) and were 29.3%, 59.2% and 11.5% for non-diabetic individuals (N = 5657).

## 4. Discussion

To our knowledge, the current study was the first that examined the relationships between home food availability and prediabetes and diabetes in adults using a nationally representative sample. The current results suggest that the availability of healthy foods such as green vegetables or fat-free/low-fat milk was inversely associated with the presence of prediabetes, whereas, a positive association between the availability of unhealthy foods such as sugary drink and prediabetes was detected adjusting for age, sex, and ethnicity. The results remained significant for green vegetables and fat-free/low-fat milk when adjusting for additional covariates including BMI and education. Furthermore, participants with high scores of overall home healthy food availability had approximately 31% decreases in odds of prediabetes compared to those with low scores, adjusting for age, sex, and ethnicity although the association was attenuated when including BMI and education as additional covariates. In contrast, no associations were detected of home food availabilities with diabetes except for fat-free/low-fat milk for which an inverse association was observed in the age, sex, and ethnicity-adjusted model.

Since the temporal relations between home food availabilities, diabetes, and prediabetes cannot be determined due to the nature of cross-sectional study design, we were not able to examine whether the associations between food availabilities and metabolic conditions (diabetes or prediabetes) were mediated by dietary intake. That being said, in the current study, we found that the significant associations of green vegetable and fat-free/low-fat milk availabilities and overall healthy food availability scores with the presence of prediabetes were independent of dietary intakes as the associations did not change substantially after further adjusting for dietary variables such as intakes of total energy, carbohydrate, sugar, fat (total) and saturated fat. The only exception was sugary drink availability with prediabetes, as adjusting for dietary components attenuated the borderline association observed in the models that was not adjusted for dietary variables (*p* = 0.06 in Model 2 vs. *p* = 0.26 in Model 3). Additionally, adjusting for dietary factors did not affect the null associations between home food availabilities and the presence of diabetes. Thus, with the estimated high rates of undiagnosed prediabetes [1], our results may suggest it is possible the individuals with prediabetes have yet to be diagnosed and were not aware of their health conditions, and therefore have not begun to make changes to their diets and home food environments. It also could be that individuals who were aware of their prediabetes status might not be fully informed about the consequences of this condition. It would be interesting and clinically meaningful to perform stratified analyses according to participants with or without a previous diagnosis of prediabetes (e.g., ever told having prediabetes) to determine whether there were differences in outcomes between those who were aware of the condition and those who were not. Unfortunately, the sample size was not sufficient (i.e., large missing data on the question “ever told you have prediabetes” in NHANES) to perform such analyses.

Our study examined whether dietary intakes of energy and major nutrients would moderate the associations between home food availabilities and the presence of metabolic diseases such as diabetes or prediabetes. We did not further examine how eating behaviors may influence the observed associations. For instance, would eating out/food prepared away from home have any influence on the current results since research has suggested having meals prepared at home more often was associated with lower risk of type 2 diabetes [24]? However, the 24-h dietary recall method is considered as the gold standard measure in nutritional epidemiologic studies and its use in NHANES to assess dietary intake is supported by expert panel discussions in “Strategies to Optimize the Impact of Nutrition Surveys and Epidemiological Studies” symposium [25]. Thus, we would assume our dietary data from multiple 24-h recalls (two recalls with an interval of 3 to 10 days) were likely to have captured the total energy and major nutrients consumed from foods/meals that were prepared away from home. Future research is warranted to use prospective cohorts to examine: (1) whether the availabilities of healthy and unhealthy food items at home are prospectively associated with the risk of prediabetes and type 2 diabetes; and (2) whether these prospective associations, if present are mediated by dietary intake or influenced by diet/nutrition-related behaviors such as frequently having meals away from home.

Previous work has extensively investigated how dietary intake of individual foods such as vegetables [26,27], low-fat dairy products [28,29,30], and sugar-sweetened beverages [31,32] as well as dietary patterns [11,12] may influence the risk of type 2 diabetes through prospective cohort studies. However, very few population-based studies were conducted to determine whether a diagnosis of type 2 diabetes or prediabetes would affect individual’s dietary behaviors and to date no studies examined how disease diagnosis would further influence the home food environment, for example, the availabilities of healthy and unhealthy foods at home. One study reported that participants who had received a diagnosis of type 2 diabetes were less likely to consume sugary drinks particularly regular soda but more likely to drink bottled waters and artificially-sweetened beverages suggesting receiving a diagnosis of diabetes might have motivated people to make healthier food choices such as choosing non-sugary beverages instead [33]. The basic concepts of several health behavior theories support the assumption that individuals are likely to change health behaviors and lifestyles after a diagnosis of a chronic health condition. For instance, according to the stage of change model [34], the onset of a serious illness could at least lead to the initial stage of change which is to acknowledge the problem. The health belief model hypothesizes that health behaviors depend on people’s beliefs about health problems [35,36]. Therefore, lifestyles are expected to improve if one recognizes the severity of the illness and clear benefits of actions. The theory of reasoned action suggests individuals’ pre-existing attitudes and behavioral intentions predict how they behave. One of the key determinants is subjective norms that refers to perceived social pressure to perform or not perform the behavior [37]. Subjective norms supporting change of behaviors after a diagnosis of a chronic health condition may play an important role for individuals to make healthy choices. In our study when comparing dietary nutrients intakes among diabetes, prediabetes and non-diabetic groups, diabetes group consumed less total energy, carbohydrate, and sugar compared to non-diabetic or prediabetes groups, while no significant differences in dietary intakes of major nutrients and total energy were observed between non-diabetic and prediabetes groups. Since this was a cross-sectional study design and based on the above behavior change theories [34,35,36,37], the current dietary results were more likely to suggest that participants with diabetes may be more aware and have made changes of their dietary intakes particularly dietary components that are more relevant to glycemic control such as dietary total energy, carbohydrate, and sugar compared to prediabetes and non-diabetic participants. These results may also to some extent support our primary findings that the associations of home food availabilities with the presence of prediabetes or diabetes were not mainly influenced by dietary factors such as total energy and major nutrient intake.

The present study has a number of strengths, including the use of a large population-based study with a nationally representative sample. Blood samples were collected to measure HbA1c levels by trained research staff to provide objective indicators for prediabetes and diabetes status. However, there were limitations to our study. One limitation was the nature of cross-sectional design of NHANES study, which prevented us from drawing causal relationships. As all prevalent case-control studies, the temporal sequence was unclear. Second, due to the self-reported measures utilized to assess food availability and dietary intakes, recall bias may occur. Third, we were not able to distinguish between type 1 and type 2 diabetes; however, the bias of misclassification would be minimum, since more than 95% of adult diabetes cases are type 2 diabetes [1]. Fourth, we collapsed the original five food availability scores into two or three categories and used odds ratios as outcome estimates in the current cross-sectional study. Thus, our outcomes could be overestimated in this regard. In addition, since the home food availability measures were only included in the 2007–2008 and 2009–2010 waves of NHANES, our results may not completely reflect the most updated situation in terms of how the availabilities of healthy and unhealthy foods at home were associated with prediabetes and diabetes. Finally, the home food availability questions in NHANES measured only five food and beverage items, which may provide a limited picture of the overall home food environment related to healthy food availability.

## 5. Conclusions

Findings from the current study suggest the availabilities of healthy foods such as green vegetables and fat-free or low-fat milk at home were inversely associated with the presence of prediabetes. Furthermore, participants with low scores of overall healthy food availability were more likely to have prediabetes. These findings were independent of dietary intakes as the results remained significant after adjusting for dietary components including total energy, carbohydrate, sugar, fat (total) and saturated fat. Thus, in addition to continuing the educational efforts made for diabetes patients, it is equally important to devote our preventative efforts to individuals who are at risk of developing type 2 diabetes, for example, those with prediabetes since the current results suggest there is a potential need to improve the home food environment related to healthy food availability for this population. Providing these individuals with useful and practical tools to monitor their household foods, food purchase habits and food choices may help to prevent and reduce the onset of type 2 diabetes. Longitudinal studies including prospective cohort studies and clinical trials are necessary to further elucidate the temporal associations between home food availability and metabolic conditions such as type 2 diabetes and prediabetes, as well as the underlying mechanisms for these associations.

## Figures and Tables

**Table 1:** Characteristics and dietary nutrient intake of study participants.

	Non-Diabetic *	Diabetes	Prediabetes
**Characteristics**			
N	5657	1197	2075
Age (year)	40.1 ± 0.3 ^a^	54.5 ± 0.5 ^b^	50.4 ± 0.4 ^c^
Sex, %			
Male	48.9	54.4	49.6
Female	51.1	45.6	50.4
Race/ethnicity, %			
Black	8.6 ^a^	17.7 ^b^	16.2 ^b^
Hispanic	13.7	16.1	14.5
White	71.3 ^a^	58.3 ^b^	62.0 ^b^
Other	6.5	8.0	7.3
Body mass index (kg/m^2^)	27.5 ± 0.1 ^a^	34.0 ± 0.3 ^b^	30.4 ± 0.2 ^c^
College graduate, %	30.8 ^a^	17.5 ^b^	22.3 ^b^
Current smoker, %	52.8 ^a^	40.7 ^b^	53.2 ^a^
**Dietary intake**			
Total energy (kcal/day)	2188 ± 19 ^a^	1947 ± 29 ^b^	2123 ± 30 ^a^
Carbohydrate (g/day)	266 ± 2 ^a^	226 ± 4 ^b^	259 ± 3 ^a^
Protein (g/day)	85.1 ± 0.8	81.8 ± 1.2	83.2 ± 1.2
Total fat (g/day)	81.7 ± 1.0	78.2 ± 1.6	81.3 ± 1.5
Saturated fat (g/day)	26.9 ± 0.4	25.3 ± 0.6	26.8 ± 0.6
Dietary fiber (g/day)	17.0 ± 0.3	16.2 ± 0.4	16.9 ± 0.3
Total sugar (g/day)	125.1 ± 2.0 ^a^	97.9 ± 4.0 ^b^	119.3± 2.0 ^a^

Note: Values are presented as weighted mean ± SE and weighted percentage (%); Values within a row with different superscript letters (a, b, c) are significantly different (*p* < 0.05). * Non-Diabetic: Individuals without the presence of diabetes or prediabetes.

**Table 2:** Associations of the availabilities of individual food items with diabetes and prediabetes.

High Availability *			Model 1 ^†^		Model 2 ^‡^		Model 3 ^§^	
	All (N)	Cases (N)	OR (95% CI) ^¶^	*p* ^¶^	OR (95% CI) ^¶^	*p* ^¶^	OR (95% CI) ^¶^	*p* ^¶^
**Diabetes (vs. non-diabetic ^ǁ^)**								
Fruits	6854	1197	0.85 (0.65–1.11)	0.21	0.89 (0.68–1.17)	0.39	0.80 (0.57–1.11)	0.17
Dark green vegetables	6854	1197	0.82 (0.65–1.04)	0.11	0.92 (0.72–1.17)	0.47	0.91 (0.69–1.21)	0.51
Fat-free/low-fat milk	6854	1197	0.80 (0.65–0.99)	0.04	0.86 (0.71–1.06)	0.16	0.85 (0.67–1.07)	0.17
Salty snacks	6854	1197	1.04 (0.85–1.26)	0.70	1.03 (0.85–1.25)	0.79	0.99 (0.80–1.22)	0.90
Sugary drinks	6854	1197	0.98 (0.79–1.21)	0.83	0.89 (0.72–1.11)	0.29	0.95 (0.76–1.20)	0.68
**Prediabetes (vs. non-diabetic ^ǁ^)**								
Fruits	7732	2075	0.90 (0.74–1.10)	0.30	0.96 (0.80–1.16)	0.68	0.88 (0.71–1.09)	0.24
Dark green vegetables	7732	2075	0.82 (0.73–0.91)	0.0006	0.86 (0.78–0.95)	0.005	0.87 (0.77–0.99)	0.03
Fat-free/low-fat milk	7732	2075	0.80 (0.65–0.89)	0.001	0.82 (0.69–0.97)	0.02	0.79 (0.67–0.93)	0.007
Salty snacks	7732	2075	0.97 (0.85–1.11)	0.62	0.98 (0.82–1.13)	0.59	0.96 (0.80–1.16)	0.68
Sugary drinks	7732	2075	1.24 (1.04–1.48)	0.02	1.17 (0.99–1.37)	0.06	1.11 (0.92–1.32)	0.26

* High availability = always/most of time available; reference group = low availability (not always/most of time available). ^†^ Model 1 was adjusted for age, sex, and race/ethnicity. ^‡^ Model 2 was adjusted for covariates in Model 1 and additionally adjusted for body mass index and education. ^§^ Model 3 was adjusted for covariates in Model 2 and additionally adjusted for dietary intakes of total energy, total carbohydrate, total sugar, total fat and total saturated fat. ^¶^ Odds ratios (ORs), 95% confidence intervals (95% CIs) and *p* values were estimated using logistic regression modeling accounting for complex survey design and sample weighting. ^ǁ^ Non-Diabetic: Individual without the presence of diabetes or prediabetes.

**Table 3:** Associations of overall home healthy food availability sores with diabetes and prediabetes.

Healthy Food Availability Score *	Participants	Model 1 ^†^		Model 2 ^‡^		Model 3 ^§^	
(Overall)	N	OR (95% CI) ^¶^	*p* ^¶^	OR (95% CI) ^¶^	*p* ^¶^	OR (95% CI) ^¶^	*p* ^¶^
**Diabetes (vs. non-diabetic ^ǁ^)**							
Overall healthy (high score)	1981	0.76 (0.54–1.07)	0.11	0.92 (0.67–1.26)	0.58	0.86 (0.60–1.25)	0.42
Borderline (medium score)	4100	0.89 (0.63–1.24)	0.46	0.98 (0.73–1.31)	0.87	0.95 (0.70–1.29)	0.71
Overall unhealthy (low score)	773	1.00		1.00		1.00	
		*p*_trend_ = 0.07		*p*_trend_ = 0.71		*p*_trend_ = 0.45	
**Prediabetes (vs. non-diabetic ^ǁ^)**							
Overall healthy (high score)	2241	0.69 (0.55–0.87)	0.003	0.80 (0.63–1.00)	0.05	0.78 (0.61–0.99)	0.04
Borderline (medium score)	4603	0.80 (0.64–1.01)	0.06	0.87 (0.71–1.06)	0.16	0.83 (0.67–1.03)	0.09
Overall unhealthy (low score)	888	1.00		1.00		1.00	
		*p*_rend_ = 0.004		*p*_trend_ = 0.07		*p*_trend_ = 0.08	

* The overall healthy food availability score was defined based on the overall availability score of healthy and unhealthy food items at home. ^†^ Model 1 was adjusted for age, sex, and race/ethnicity. ^‡^ Model 2 was adjusted for covariates in Model 1 and additionally adjusted for body mass index and education. ^§^ Model 3 was adjusted for covariates in Model 2 and additionally adjusted for dietary intakes of total energy, total carbohydrate, total sugar, total fat and total saturated fat. ^¶^ Odds ratios (ORs), 95% confidence intervals (95% CIs) and *p* values were estimated using logistic regression modeling accounting for complex survey design and sample weighting. ^ǁ^ Non-Diabetic: Individual without the presence of diabetes or prediabetes.
